# AcTor, a novel mTOR stimulator, potentiates ixazomib for the treatment of acute myeloid leukemia

**DOI:** 10.21203/rs.3.rs-9405584/v1

**Published:** 2026-04-23

**Authors:** Shakti P Pattanayak, Odai Darawshi, Omid Hajihassani, Jordan M Winter, Nicole Weiler, Melanie Ott, Florian Rothweiler, Jindrich Cinatl, Martin Michaelis, Daniel J Lindner, Thomas D Green, Polina Krassovskaia, Raphael T Aruleba, Kelsey H Fisher-Wellman, Jason A Mears, David Wald, Leif A Eriksson, Boaz Tirosh

**Affiliations:** Case Western Reserve University School of Medicine; Medical University of South Carolina; University Hospital; University Hospital; Dr Petra Joh Research Institute; Dr Petra Joh Research Institute; Dr Petra Joh Research Institute; Dr Petra Joh Research Institute; University of Kent; Cleveland Clinic; Wake Forest University School of Medicine; Wake Forest University School of Medicine; Wake Forest University School of Medicine; Wake Forest University School of Medicine; Case Western Reserve University; Case Western Reserve University; University of Gothenburg; Case Western Reserve University School of Medicine

## Abstract

**Background:**

mTORC1 activity is oncogenic. However, in the presence of chemotherapy, suppression of mTORC1 is cytoprotective. mTOR suppression requires an intact tuberous sclerosis complex (TSC), composed of TSC1, TSC2 and TBC1D7. Small molecules that activate mTOR by blocking the TSC are lacking.

**Methods:**

We applied *in silico* docking and medicinal chemistry to generate AcTor, a potential first-of-its-kind TSC2 inhibitor. Because inhibition of TSC2 results in increased sensitivity to proteasome inhibitors, we combined AcTor and the proteasome inhibitor ixazomib (IXZ) in various cancer cell types.

**Results:**

Potentiation of cytotoxic activity of IXZ by AcTor was observed across multiple acute myeloid leukemia (AML) cell lines and primary patient samples. The combination triggered a collapse of mitochondrial respiratory capacity, loss of mitochondrial membrane potential, accumulation of ROS and apoptosis. These attributes increased in drug-resistant AML. Transcriptomic profiling revealed that AcTor alone induced anabolic and oxidative phosphorylation programs, whereas AcTor/IXZ redirected the signaling towards stress-associated and pro-apoptotic transcriptional states, including a p53 pathway signature. *In vivo* studies revealed reduction in AML burden, depletion of blasts and of leukemic stem cells, and retention of activity upon relapse. AcTor/IXZ was equally potent in a *TP53*-mutated patient-derived xenograft model, exceeding the efficacy of standard-of-care.

**Conclusions:**

As a TSC2 inhibitor, AcTor should not be used alone in cancer. When combined with proteasome inhibitors, the pharmacodynamics of AcTor shifts towards the development of a mitochondrial catastrophe in AML, which is durable, broad range, agnostic to *TP53* mutations and to the acquisition of resistance to common clinical anti-AML drugs.

## Introduction

Acute myeloid leukemia (AML) is a consequence of transformed myeloid precursor cells in bone marrow, resulting in an accumulation of abnormal, immature myeloid cells. AML is driven by leukemic stem cells (LSCs), which proliferate, gradually overtake the bone marrow, giving rise to AML blast cells that are released to the bloodstream [1]. Inevitably, over time, an irreversible bone marrow failure ensues. AML affects both children and adults. While children respond well to allogeneic stem cell transplantation procedures and high dose chemotherapy [2], most adults do not. Hence, prognosis of AML in adults is dire with five-year survival of less than 5% in over 60-year-old patients. One of the indicators of poor prognosis is loss-of-function mutations in the *TP53* gene that encodes the tumor suppressor p53 protein [3]. Because functional p53 curtails AML proliferation and promotes response to therapy, *TP53*-mutated myeloid neoplasms are highly aggressive with a dismal overall survival of less than a year [4]. Mutations in *TP53* are found in less than 10% of patients with newly diagnosed AML; however, they increase remarkably to approximately 40% in relapsed AML [5, 6]. These tumors are currently treated with venetoclax, a B-Cell lymphoma-2 protein inhibitor in combination with hypomethylation agents [6, 7]. However, inactivation of *TP53* is a main driver of resistance to venetoclax [8]. Hence, only a modest increase in overall survival is obtained with these drugs. Other therapies, biological, cell-based and small molecules, have shown a limited response [9]. Thus, novel therapies for AML in general and *TP53-*mutated AML in particular are urgently needed.

The proteasome inhibitor (PI) bortezomib was introduced for the treatment of multiple myeloma (MM) over two decades ago. Since then, two additional PIs were approved for MM, carfilzomib and ixazomib (IXZ). Bortezomib and carfilzomib are given by injections. IXZ is orally available, given in capsules [10]. The underlying reasons for the exquisite sensitivity of MM to PIs are not fully understood. The prevailing paradigm invokes the combination of accumulation of unfolded proteins in the endoplasmic reticulum (referred to as ER stress) and in the cytoplasm [11], together with the stabilization of the pro-apoptotic protein JNK [12] and the inhibitor of NF-κB [13]. Importantly, although p53 is subjected to rapid proteasomal degradation, and PIs stabilize p53, the clinical efficacy of PIs in MM is similar in *TP53-*mutated and non-mutated MM [14–16]. Extensive efforts have been invested in trying to find additional indications for PIs. Despite similarities between MM and AML with respect to protein synthesis and degradation, PIs fell short to significantly extend the overall survival in AML [17]. We reasoned that the tolerability of PIs in elder patients [18] and their mechanism of action should improve therapy also of *TP53*-mutated AML, if a further boost in activity is achieved.

mTOR resides in either of two complexes: mTORC1 and mTORC2, which dictate specificity and regulation. mTORC1 promotes anabolic programs, inhibits autophagy, and induces the biosynthesis of proteins, lipids, and nucleic acids. These activities promote cell growth and survival and play a role in oncogenesis [19]. However, despite promising preclinical data, the clinical effects of mTOR inhibitors have thus far been disappointing [20], in part due to prosurvival roles of mTORC1 suppression in solid [21] and hematological tumors [22] in response to therapy [23]. mTORC1 is negatively controlled by the tuberous sclerosis complex (TSC). TSC2 is the catalytic subunit of the complex, which operates as a GTPase-activating protein (GAP) for Rheb, an essential G protein for mTORC1 activation [24]. Deletion of TSC2 leads to the most potent and direct hyperactivation of mTORC1, and to a disconnection from upstream regulation [22, 25, 26]. We recently found that an early response to PIs is a strong suppression of mTORC1 activity, and MM deficient for TSC2 acquires sensitivity to PIs[22]. Surprisingly, the mechanism of death was not associated with ER stress, but rather a mitochondrial dysfunction, which is orthogonal to DNA damage, the standard of care in AML. Here, we examined the activity of a new TSC2 inhibitor, AcTor. AcTor enhanced the cytotoxicity of IXZ by 15-fold across multiple acute myeloid leukemia (AML) cell lines irrespective of mutations in *TP53*. *In vivo*, AcTor/IXZ combination improved the survival of mice engrafted with *TP53* intact and mutated AML and maintained potency after relapse. We propose AcTor as an enhancer of PI activity, which can be leveraged for AML therapy.

## Material and Methods

All materials and method information is provided in the supplemental material.

## Results

### Design of AcTor:

**Figure 1:**
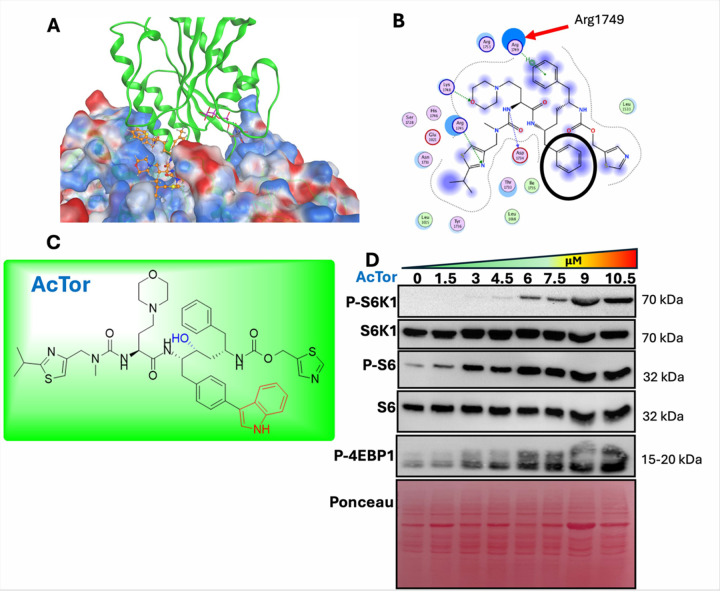
Rationale design of AcTor as a TSC2 inhibitor. **(A)** *In silico*docking of cobicistat to the model of TSC2 and Rheb. Cobicistat mostly binds to TSC2. **(B)** A 2D projection of cobicistat binding demonstrates the proximity to the catalytic residue Arg1749 of TSC2 and the protrusion of the phenyl group into Rheb, circled in black. **(C)** AcTor is a modified cobicistat. **(D)** RPMI 8226 cells were treated for 24 h with the indicated concentrations of AcTor. Total cell lysates were analyzed by immunoblotting. mTORC1 activity was assessed by P-S6K1, P-S6 and P-4EBP1 levels.

Protein-protein interactions rely on multiple contacts, and small molecules are typically inefficient in dissociating protein complexes. However, binding of small molecules can modulate the configuration of the complex, affecting activity [27]. We identified a gap within the interphase of TSC2 and Rheb that can accommodate a small molecule [28]. The proximity to the catalytic site of TSC2 suggested that a small molecule could alter mTORC1 activity. We docked *in silico* all clinically approved drugs to this interspace. Several of the top binders were chelating agents carrying several carboxylic acids, charged phosphates, or quaternary amine groups. These molecules were discarded, given their low cellular permeability. Other molecules, albeit obtaining a high free energy of binding, were found to interact deeper inside the binding area and thus are not likely to impede TSC2/Rheb interaction. The cytochrome P450 inhibitor cobicistat stood out as the only potential hit. We pursued it owing to drug-like structure and oral bioavailability (Fig. 1A). A two-dimensional projection of cobicistat binding onto the TSC2 binding pocket suggests proximity to Arginine1749 in TSC2, a critical residue for TSC2 catalytic activity [28]. The model shows that the morpholine group of cobicistat points to TSC2 and the phenyl group, circled, protrudes towards Rheb. Cobicistat formed multiple interactions with the key residues and covered/blocked essentially the entire recognition area (Fig. 1B). However, when tested in RPMI 8226 cells, a reduction in mTORC1 activity was observed, assessed by the phosphorylation level of S6. Reduction in activity occurred in NPRL2 KO and was not observed in TSC2 KO RPMI 8226 cells (**Fig. S1A**). Based on the *in silico* model, we predicted that modifications of the phenyl group of cobicistat with bulky moieties may convert it to an mTORC1 activator. To identify potential modifications, the solvent-exposed phenyl ring was targeted using R-group replacement. Among the fragments explored, 5-indole bound in para position provided the best docking score, ~1kcal/mol better than the original compound itself. The indole moiety furthermore orients into the cavity of the GAP domain of TSC2, hindering Rheb from entering the binding site. Following optimization of a synthetic scheme, AcTor was prepared. In color are the modifications that were introduced to cobicistat (Fig. 1C).

AcTor activates mTORC1 in the presence of IXZ and potentiates IXZ activity across multiple AML cell lines:

The similarity of AcTor to cobicistat suggested that it too may be orally available. We therefore studied its activity in the MM cell line RPMI 8226 together with the orally available PI, ixazomib (IXZ). AcTor induced the activity of mTORC1 in a concentration-dependent manner, plateauing around 10 μM (Fig. 1D). At this concentration, AcTor prevented the suppression of mTORC1 activity by IXZ, without affecting the levels of ubiquitinated proteins (**Fig. S1B, S1C**). AcTor alone mildly compromised the viability of RPMI 8226 after prolonged incubation, an effect that was not observed with cobicistat (**Fig. S1D**). When combined with 20 nM of IXZ for 24 h, viability was compromised more than in the presence of IXZ alone. This was partially rescued by the addition of the mTOR inhibitor Torin-1 (**Fig. S1E**). Immunoprecipitation studies suggested that the interaction between TSC2 and Rheb was not prevented by AcTor (**Fig. S2A**), Rheb•GTP levels increased (**Fig. S2B**). To assess binding of AcTor to TSC2 indirectly, we performed two assays: thermostability and drug affinity responsive target stability (DARTS) [29]. The first assesses the improvement in solubility following drug binding; the second tests protection against protease digestion. The addition of AcTor improved the solubility of TSC2 at 57°C compared to 61°C for the DMSO control (**Fig. S2C, S2D**). Utilizing this assay, the stabilization of TSC2 by AcTor was maximal at approximately 10 μM (**Fig. S2E**), in agreement with mTORC1 activity. By using the protease pronase at low amounts, we found that AcTor protected TSC2 from degradation (**Fig. S2F**). These findings suggest that AcTor binds to TSC2 and reduces its ability to suppress Rheb.

**Figure 2:**
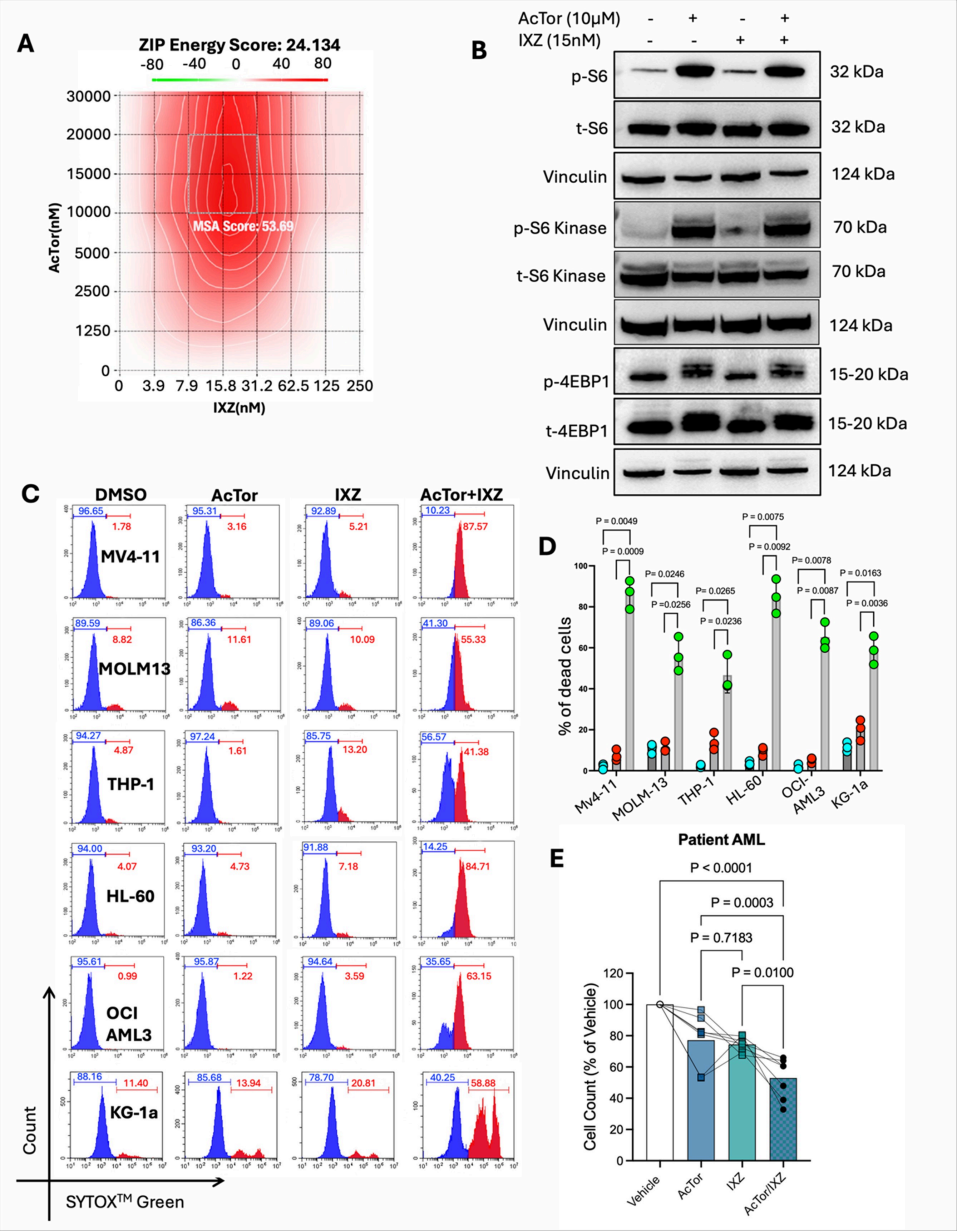
AcTor and IXZ synergize in AML cells. **(A)** MV4–11 cells were treated with 7 different concentrations of AcTor and 7 different concentrations of IXZ at all possible combinations. 24 h later, viability was assessed by CellTiter-Glo. A synergy score was calculated using the SynergyFinder tool. **(B)** MV4–11 cells were treated with DMSO, AcTor and/or IXZ at the indicated concentrations for 24 h. Live cells were separated by a lymphoprep gradient, and total cell lysates were analyzed by immunoblotting for mTORC1 activity. Shown is a typical result of three independent experiments. **(C)** Six different AML cell lines were treated with DMSO, AcTor (10 μM), IXZ (15 nM) or AcTor/IXZ combination for 24 h. Viability was measured by flow cytometry using Sytox Green as a viable dye. Shown is a representative result of three independent repetitions. **(D)** Quantification of three repetitions comparing IXZ to AcTor/IXZ. **(E)** Bone marrow aspirates of AML patients were grown *ex vivo* for a week and then treated with DMSO, AcTor, IXZ and combination for 72 h. Total viable cell counts were measured by ViCell and plotted as a percentage than the DMSO control. Lines connect the individual samples across treatments. Data are presented as mean ± SD from n=3 independent biological replicates. Statistical significance was determined using Brown-Frosythe and Welch one-way ANOVA followed by Dunnett’s T3 multiple comparisons test. Calculated *P* values are indicated in the graphs.

To test whether the enhanced activity of IXZ by AcTor also applies to AML cells, MV4–11 AML cells, which harbor the MLL-AF4 fusion gene and a FLT3 activation mutation [30], were plated in a matrix of concentrations of AcTor and IXZ, and viability was measured 24 h later by CellTiter-Glo. To quantify synergism, we applied the SynergyFinder tool [31, 32]. A ZIP energy score of over 5 is indicative of synergism. We calculated a score of 24. The peak of the synergism map was at approximately 15 nM of IXZ and 10 μM of AcTor (Fig. 2A, **Fig. S3**). When surviving cells were analyzed by immunoblotting following treatment at these concentrations, mTORC1 output (P-S6K1, P-S6, P-4EBP1) was higher in the presence of AcTor/IXZ compared to IXZ alone (Fig. 2B). We analyzed the activity of AcTor/IXZ at these concentrations in multiple AML cell lines by flow cytometry, using SYTOX green as a vital dye. A strong synergism was observed in all tested cells (Fig. 2C), quantified in Fig. 2D. Of note, in MV4–11 cells, a concentration as high as 250 nM of IXZ did not achieve a similar cytotoxic activity as 15 nM IXZ in the combination after 24 h (**Fig. S4A**). These data indicate potentiation of IXZ activity by more than 15-fold. *Ex vivo*, primary AML cells were sensitive to AcTor alone and more sensitive to the combination (Fig. 2E). Cobicistat, on the other hand, did not enhance the activity of IXZ, even at 30 μM (**Fig. S4B,C**). To exclude that AcTor operates through cytochrome P450 inhibition, we applied ketoconazole, a potent cytochrome P450 inhibitor with an IC_50_ of less than 1 μM [37]. Ketoconazole did not enhance the activity of IXZ, even at much higher concentrations than its IC_50_. Only when AcTor was added on top of ketoconazole/IXZ, enhanced activity was observed (**Fig. S4D,E**). We conclude that AcTor is a different pharmacological entity than Cobicistat and potentiates IXZ activity by preventing mTOR suppression.

### AcTor/IXZ treatment causes mitochondrial dysfunction in AML:

**Figure 3:**
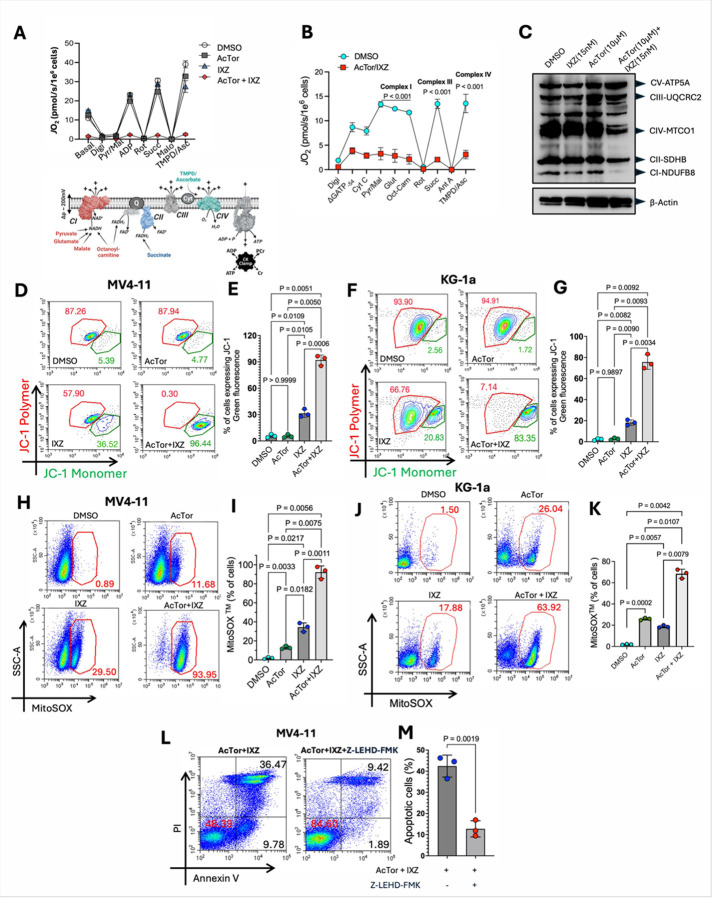
The combination of AcTor/IXZ generates mitochondrial damage. **(A)** MV4–11 were treated for 24 h with 5 μM of AcTor, 15 nM of IXZ or both. Then, cells were permeabilized with digitonin (0.02 mg/ml) and respiration was assessed in response to the indicated additions. Data normalized to viable cell count. **(B)** KG-1a cells treated for 24hrs with DMSO or the combination of 10 μM of AcTor and 15 nM of IXZ. Cells were permeabilized with digitonin (0.02 mg/ml) and respiration was assessed in response to the indicated additions. Data normalized to viable cell count. **(C)** Total lysates from live MV4–11 cells after treatment were immunoblotted with a total OXPHOS antibody cocktail detecting subunits of complexes I–V. The combination of Actor and IXZ led to a marked reduction in complexes I, II, and IV, indicating impaired electron transport chain integrity, while complex V (ATP synthase) remained largely unaffected. **(D- G)** MV4–11 and KG-1a cells were treated for 24 h with DMSO, AcTor (10 μM), IXZ (15 nM) or AcTor/IXZ and stained with JC-1 for mitochondrial membrane potential analysis. Shown is a representative experiment for each cell type and the quantification of three independent repetitions. **(H-K)**Following treatments, cells were stained with MitoSox (1 μM) for 20 min and analyzed by flow cytometry. Shown is a representative experiment for each cell type and the quantification of three independent repetitions. **(L** and**M)** MV4–11 cells were treated with AcTor/IXZ for 24 h in the presence and absence of the caspase 9 inhibitor Z-LEHD-fmk (25 μM). Flow cytometry was performed with Annexin V/PI to determine apoptosis. Shown is a representative result and the quantification of three independent repetitions. Data are presented as mean ± SD from n=3 independent replicates. Statistical significance was determined using Brown-Frosythe and Welch one-way ANOVA followed by Dunnett’s T3 multiple comparisons test. Comparison of two groups (AcTor/IXZ vs AcTor/IXZ/Z-LEHD-FMK) was done using a two-tailed unpaired t test. Calculated *P* values are indicated in the graphs.

TSC2 KO cells develop mitochondrial dysfunction in the presence of IXZ [22]. To examine whether AcTor exerts a similar function, we measured the effect of AcTor on mitochondrial respiration in the presence and absence of IXZ by Seahorse. AcTor alone did not significantly affect the oxygen consumption rate of MV4–11 cells. A small reduction was observed by IXZ. When AcTor was combined with IXZ, a complete shutdown of mitochondrial respiration was recorded (**Fig. S5A**). To ensure that the lack of mitochondrial activity is the cause of cell death, we applied a mitochondria diagnostic assay in which the cells are permeabilized with digitonin and the mitochondria are sequentially energized with carbon substrates. A 24 h exposure of MV4–11 cells to AcTor/IXZ was sufficient to nearly eliminate mitochondrial respiration regardless of added carbon sources (Fig. 3A). Sequential additions of substrates of the electron transport chain complexes did not restore oxygen consumption (Fig. 3B). This implies that the combination of AcTor and IXZ induces a bioenergetic crisis. Analysis of the expression of subunits of each of the ETC complexes in the remaining live cells after 24 h of treatment with AcTor/IXZ demonstrated a compound reduction in expression (Fig. 3C). Consistently, a complete loss of the mitochondrial membrane potential was measured by JC-1 for both MV4–11 (Fig. 3D and E) and KG-1a cells (Fig. 3F and G). This was associated with the induction of mitochondrial ROS, measured by MitoSOX (Fig. 3H–K). Total ROS levels were also increased but not to the same extent (**Fig. S5B**), suggesting that the source of ROS is primarily mitochondrial. The mitochondrial damage induced apoptosis, demonstrated by PARP1 cleavage (**Fig. S5C**) and Annexin V/propidium iodide (PI). Viability of the MV4–11 and KG-1a cells was improved by inclusion of the pan-caspase inhibitor zVAD-fmk (**Fig. S5D-G**). To validate that the mitochondrial dysfunction is related to TSC2 inhibition in the presence of IXZ, we suppressed TSC2 expression in two AML cell lines, MV4–11 and THP-1 (**Fig. S6A**). In both TSC2 suppressed cells, IXZ alone compromised cell viability. The addition of AcTor did not significantly enhance IXZ activity (**Fig. S6B-6E**). This was reflected in the loss of mitochondrial membrane potential by IXZ alone (**Fig. S6F-6I**). These findings are consistent with AcTor operating primarily by inhibiting TSC2. We then used two mitochondrial dyes to image mitochondria content and function. MitoTracker red accumulates in the mitochondria in a membrane potential-dependent manner, while MitoTracker green binding is insensitive to membrane potential, and serves as a readout of mitochondria content. MV4–11 cells treated for 24 h with AcTor, IXZ, or AcTor + IXZ displayed a similar mitochondria content (green signal, **Fig. S7A**). The mitochondrial potential was induced by AcTor and reduced when AcTor and IXZ were combined relative to controls (red signal, **Fig. S7B**). Of note, an increase in mitochondrial potential has been reported for TSC2 silenced cells [38]. To assess whether the mitochondrial stress is the primary inducer of cell death, we blocked caspase 9 during AcTor/IXZ treatment with Z-LEHD-FMK. Percentage of apoptotic cells was significantly reduced (Fig. 3K and L). These data indicate that AcTor/IXZ combination creates irreparable damage to mitochondrial electron transport chain (ETC). Importantly, since ROS induces differentiation of AML LSCs [39],, the burst in ROS may be an advantageous pharmacodynamic effect of AcTor/IXZ treatment.

### AcTor/IXZ treatment gains potency in drug-resistant AML:

**Figure 4:**
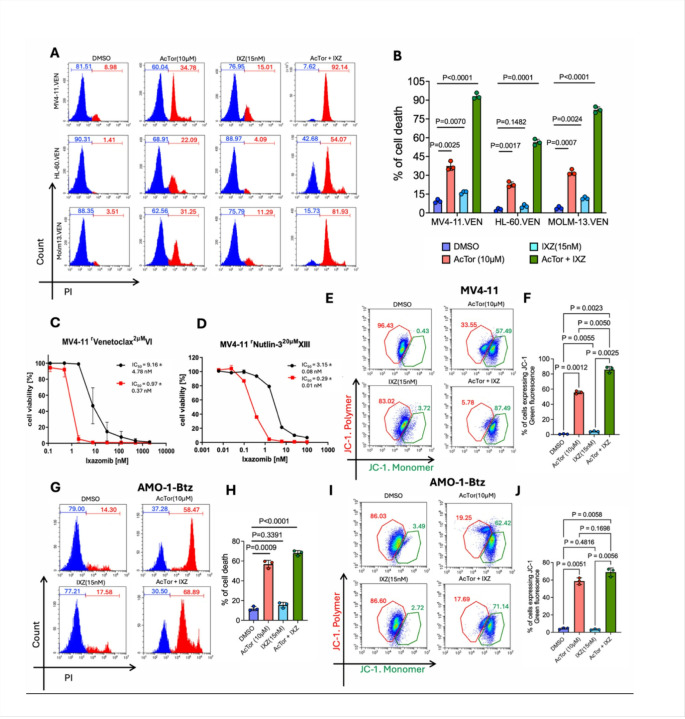
AcTor/IXZ treatment gains potency in drug-resistant AML. **(A-B**) Venetoclax-resistant AML cells (MV4–11, HL-60, and Molm-13) were treated with DMSO, AcTor, IXZ or the combination for 24 h, stained with PI and analyzed by flow cytometry. Experiments were done in three independent replicates. **(C-D)** Similar as in **A**, only after 20 h, cells were stained with JC-1. **(E** and **F)** Shown are dose–response curves and IC_50_ values for IXZ, measured by CellTiter-Glo, in the presence and absence of AcTor (2 μM) in a venetoclax-resistant MV4–11 clone and a Nutlin-3-resistant clone, each of which is *TP53* mutant. (**G** and **H**) Bortezomib-resistant AMO-1 multiple myeloma cells were treated and analyzed as in A. (**I** and **J**) Analysis of mitochondrial membrane potential by JC-1 and quantification of three repetitions. Statistical significance was determined using Brown-Frosythe and Welch one-way ANOVA followed by Dunnett’s T3 multiple comparisons test. Calculated *P* values are indicated in the graphs.

Relapse is common in adult AML, and patients who relapse are frequently treated with the BCL2 inhibitor Venetoclax. However, resistance inevitably develops employing multiple mechanisms, including metabolic reprogramming characterized by increased mitochondrial ATP hydrolysis [40, 41]. To examine if AcTor/IXZ efficacy is effective in venetoclax-resistant AML, we used three resistant AML cell lines: MV4–11, HL60 and Molm13. In all three models, AcTor alone compromised cell viability, and this effect was further enhanced when AcTor was combined with IXZ (Fig. 4A, 4B). Consistent with these findings, analysis of mitochondrial membrane potential in the resistant MV4–11 cells showed that treatment with AcTor alone generated a loss in more than half of the cells, an effect that was further increased in presence of IXZ (Fig. 4C, 4D). Next, we measured the IC_50_ of IXZ in the presence and absence of AcTor. To this end AcTor concentrations were reduced to 2 μM to avoid the confounding effect of its activity as a single agent. In the presence of AcTor, IC_50_ of IXZ was reduced by more than 10-fold to a sub-nanomolar level in a venetoclax-resistant MV4–11 clone (Fig. 4E). Almost half of relapsed AML is mutated for the *TP53*, and these patients survive on average less than a year [42]. MV4–11 has a low frequency of *TP53*-mutant cells [43]. We have established a *TP53*-mutant MV4–11 subclone by selection with the p53 stabilizing drug nutlin-3 [44]. When tested in these cells, AcTor reduced the IC_50_ of IXZ also by approximately 10 fold (Fig. 4F). These results indicate that the AcTor/IXZ combination gains activity in AML cells that have acquired resistance to venetoclax, most likely due to the remodeling in metabolism. We then asked whether AcTor could also maintain its activity in the setting of resistance to proteasome inhibitors. We approached this in the bortezomib-resistant AMO-1 multiple myeloma cells. Similar to venetoclax-resistant AML, AcTor alone compromised cell viability (Fig. 4G, 4H) and induced a significant loss of mitochondria membrane potential (Fig. 4I, 4J). In these cells, IXZ alone minimally affected survival, indicating that resistance to bortezomib confers resistance to IXZ. These data suggest that AcTor/IXZ can be effective in relapsed, resistant AML.

### Gene expression induced by AcTor alone does not overlap with that of AcTor/IXZ:

**Figure 5:**
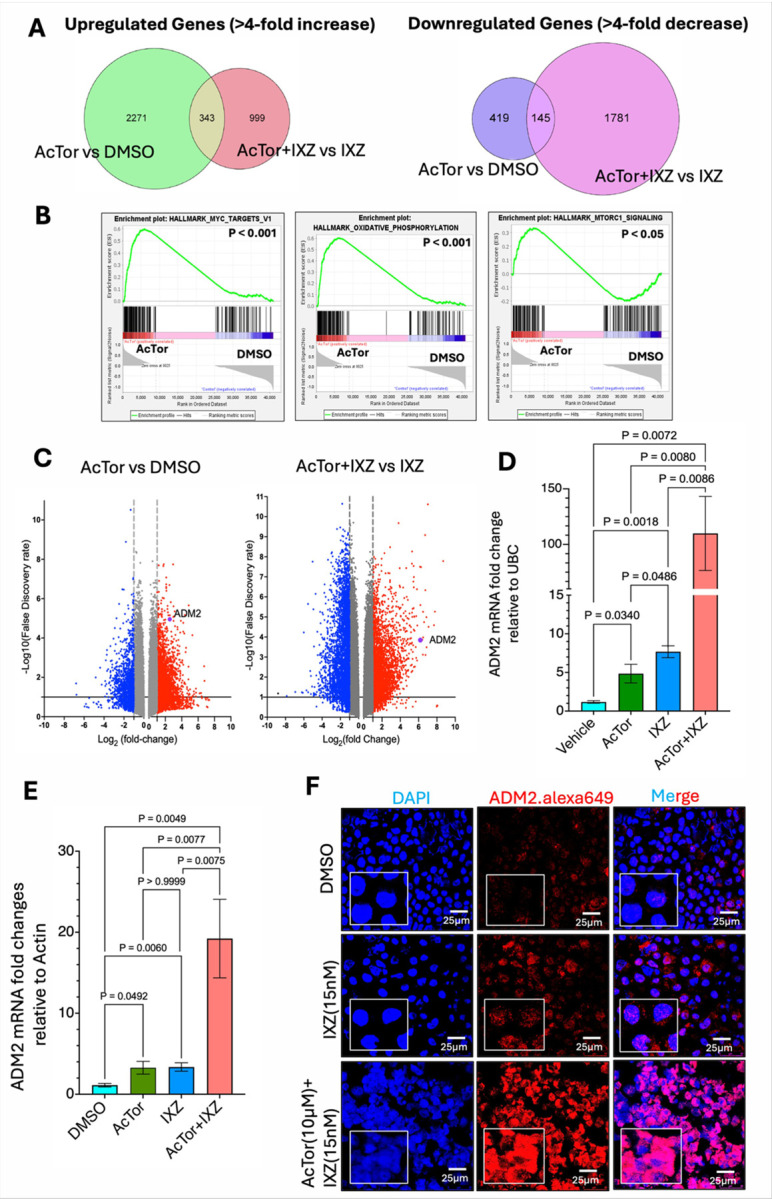
AcTor/IXZ activates a stress program that is different from AcTor alone. **(A)** MV4–11 were treated with DMSO, AcTor (10 μM), IXZ (15 nM) or AcTor/IXZ for 24 h. Live cells were separated by a lymphoprep gradient, and RNA was extracted and sequenced. Shown are Venn diagrams of the upregulated and downregulated genes between AcTor vs DMSO and AcTor/IXZ vs IXZ. **(B)** Analysis of differentially expressed genes of AcTor vs DMSO and AcTor/IXZ vs IXZ. **(C, D)**ADM2 is induced by AcTor/IXZ at the mRNA level in MV4–11 and in KG-1a cells (**E**), and at the protein level in MV4–11 **(F)**, assessed by immunofluorescence. Data are presented as mean ± SD from n=3 independent biological replicates. Statistical significance was determined using Brown-Frosythe and Welch one-way ANOVA followed by Dunnett’s T3 multiple comparisons test. Calculated *P* values are indicated in the graphs.

We compared the transcriptome of MV4–11 cells treated with AcTor versus DMSO control to AcTor/IXZ versus IXZ. AcTor induced 2271 genes relative to DMSO. Only 343 of these overlapped with the induced genes of AcTor/IXZ versus IXZ. A similar small overlap was observed for the downregulated genes (Fig. 5A). Analysis of the differentially expressed genes indicated upregulation of Myc targets, genes of the oxidative phosphorylation pathway, and mTORC1 signaling by AcTor (Fig. 5B). These signatures were abolished when AcTor/IXZ was compared to IXZ. Instead, signatures of stress signaling and proapoptotic pathways emerged, such as a p53 signature (**Fig. S8A**). Comparisons of AcTor alone vs AcTor/IXZ demonstrate a diversion from the classical mTOR effect on anabolic programs and cell proliferation (**Fig. S8B**). Connectivity genes in AcTor vs DMSO were associated with proliferation and survival, such as tyrosine kinase receptor signaling and promotion of mitochondrial activity, while the connectivity genes in AcTor/IXZ vs IXZ were mostly stress-inducing genes (**Fig. S8C**). Taken together, although AcTor/IXZ elevates mTORC1 activity, the downstream program is deviated into stress-induced cell death pathways. When analyzing the volcano plots of AcTor/IXZ vs IXZ, we noticed that the induction of Adrenomedullin 2 (ADM2) by AcTor/IXZ combination (Fig. 5C). Since ADM2 is a secreted protein [45], we pursued it as a biomarker for the treatment. qPCR analysis confirmed the induction of ADM2 at the mRNA level in MV4–11 (Fig. 5D) and KG-1a cells (Fig. 5E), and by immunofluorescence at the protein level (Fig. 5F).

#### AcTor and IXZ synergize in vivo to eradicate AML blasts and AML stem cells and the treatment maintains potency after relapse

Cobicistat is given to mice at a dose of 25 mg/kg [46]. With the adjustment of the higher mw of AcTor, we assessed 30 mg/kg as a therapeutic dose. We conducted a thorough toxicity analysis that includes histology of the different organs, assessment of serum transaminase levels and complete blood counts. Repetitive daily doses of AcTor at 30 mg/kg for three weeks did not show signs of toxicity. We did not observe overt toxicities following a single dose of AcTor of up to 300 mg/kg. IXZ at doses above 2 mg/kg i.p. every other day were not tolerated. Every other day doses of up to 1 mg/kg did not show acute toxicity. We tested the combination of AcTor (30 mg/kg) and IXZ (1 mg/kg) for three weeks in C57BL/6J mice. Mice continued to gain weight, splenic B cells and T cells were not significantly affected, blood counts were normal, and no pathologies were observed in the different organs (**Fig. S9**). To assess concerns related to chemotherapy-induced immunosuppression, we analyzed the effect of AcTor/IXZ on hematopoietic stem cells (HSCs) in C57BL/6J mice. We observed a 30% reduction in the number of bone marrow HSCs (**Fig. S10**). This reduction, though significant, did not lead to a measurable reduction in peripheral B or T lymphocytes, suggesting it should not compromise immune functions.

**Figure 6:**
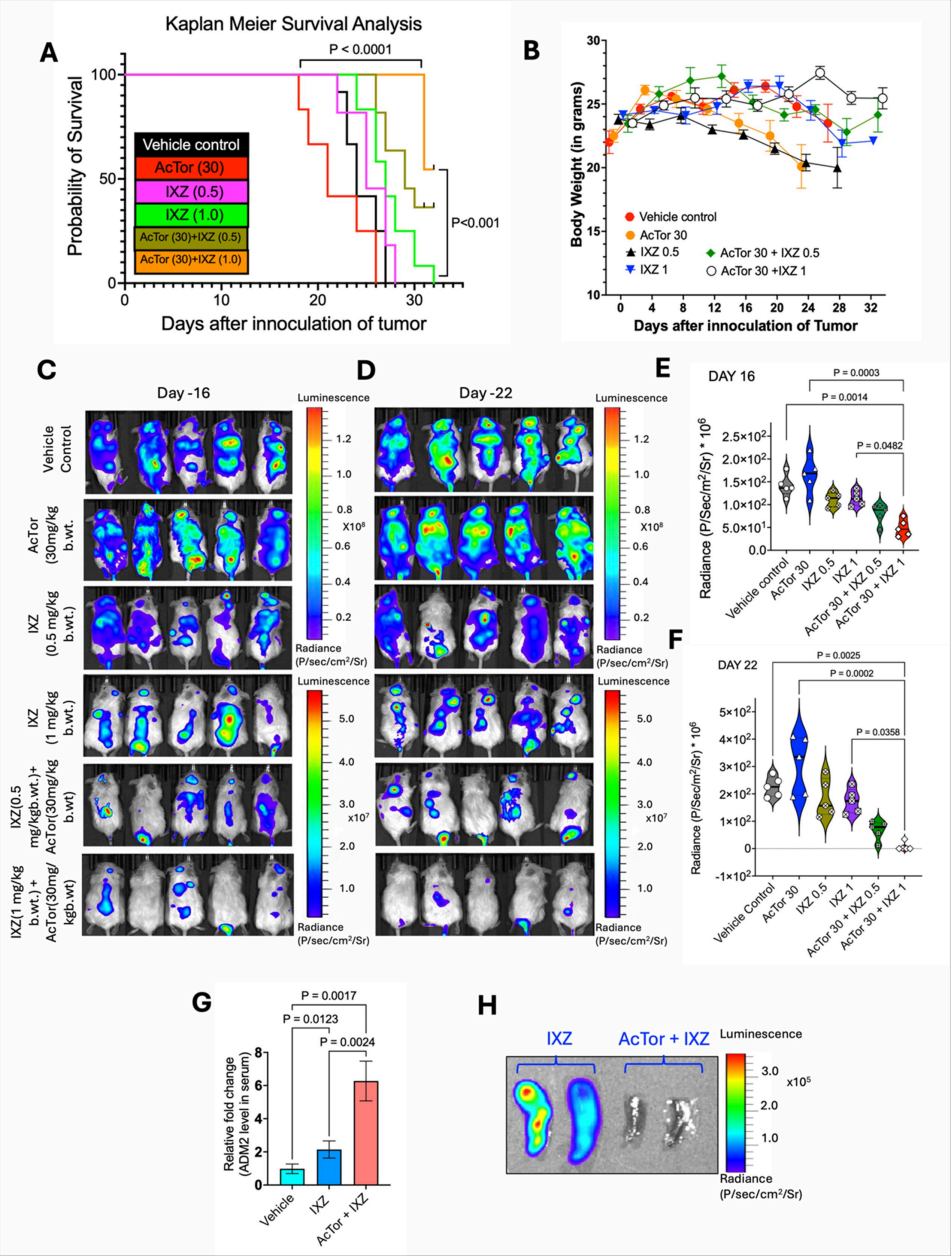
Treatment with AcTor/IXZ for three weeks improves survival in an aggressive in vivo model of AML. **(A)** NSG mice were engrafted with MV4–11-luciferase cells. Mice were treated for three weeks as indicated. Survival curves were generated using the Kaplan-Meier method and compared using the log-rank (Mantel-Cox) test. For experiments involving multiple groups during survival analysis, pairwise comparisons were adjusted using the Holm- Šídák method (n = 12). **(B)** Measurement of body weight. **(C, D)** Total body luminescence of individual mice and the quantification of the signal (**E,F**) taken at days 16 and 22 postinoculation. Statistical comparisons among groups were performed using the non-parametric Kruskal-Wallis test, followed by Dunn’s multiple comparisons test (n =5). **(G)** Relative levels of ADM2 in the serum of treated mice analyzed at day 16. **(H)** Shown are two spleens of IXZ (1 mg/kg) and the AcTor (30 mg/kg)/IXZ (1 mg/kg) cohorts analyzed at the end of the experiment by luminescence. Statistical significance was determined using Brown-Forsythe and Welch one-way ANOVA followed by Dunnett’s T3 multiple comparisons test. Calculated *P* values are indicated in the graphs

Engraftment of NSG mice with MV4–11 cells is an aggressive AML model, which cripples the mice within 2–3 weeks [47]. One million MV4–11 cells that stably express luciferase were injected intravenously. Because of model aggressiveness, three days after the challenge, we initiated treatment i.p. with vehicle, AcTor, IXZ, or AcTor + IXZ every other day. Tumor burden was assessed by total body luminescence. Following three weeks, most mice in the vehicle control and the AcTor alone group succumbed. The best survival was obtained with the combination of 30 mg/kg of AcTor with 1 mg/kg of IXZ (Fig. 6A). Of note, AcTor alone seemed to modestly accelerate disease progression, consistent with the positive effect on mTORC1. Hence, a treatment with AcTor alone should be avoided. The positive effect of the combination was apparent in the mouse weight (Fig. 6B). To assess tumor burden, we randomly selected 5 animals for total body luminescence analysis. At day 16, prior to mouse death, tumor burden was lowest in the AcTor with high dose IXZ (Fig. 6C and E). This trend continued to day22 after inoculation, when only 5 mice were left in the AcTor alone treated group (Fig. 6D and F). At day 22 prior to luminol injection, we bled 5 mice from vehicle, IXZ (1 mg/kg) and AcTor/IXZ cohorts or ADM2 analysis in the serum. Levels were higher in the AcTor/IXZ group (Fig. 6G), suggesting that serum ADM2 can be a biomarker for response. At day 32, when the 2 animals of IXZ only cohort were left and appeared very sick, we administered the luciferin and terminated the experiment. The spleens of the two mice and two of the AcTor/IXZ group were imaged ex vivo. Hardly any signal was observed for the AcTpr/IXZ treated mice (Fig. 6H). We conclude that AcTor potentiates IXZ for the treatment of a highly *in vivo* aggressive AML model.

**Figure 7:**
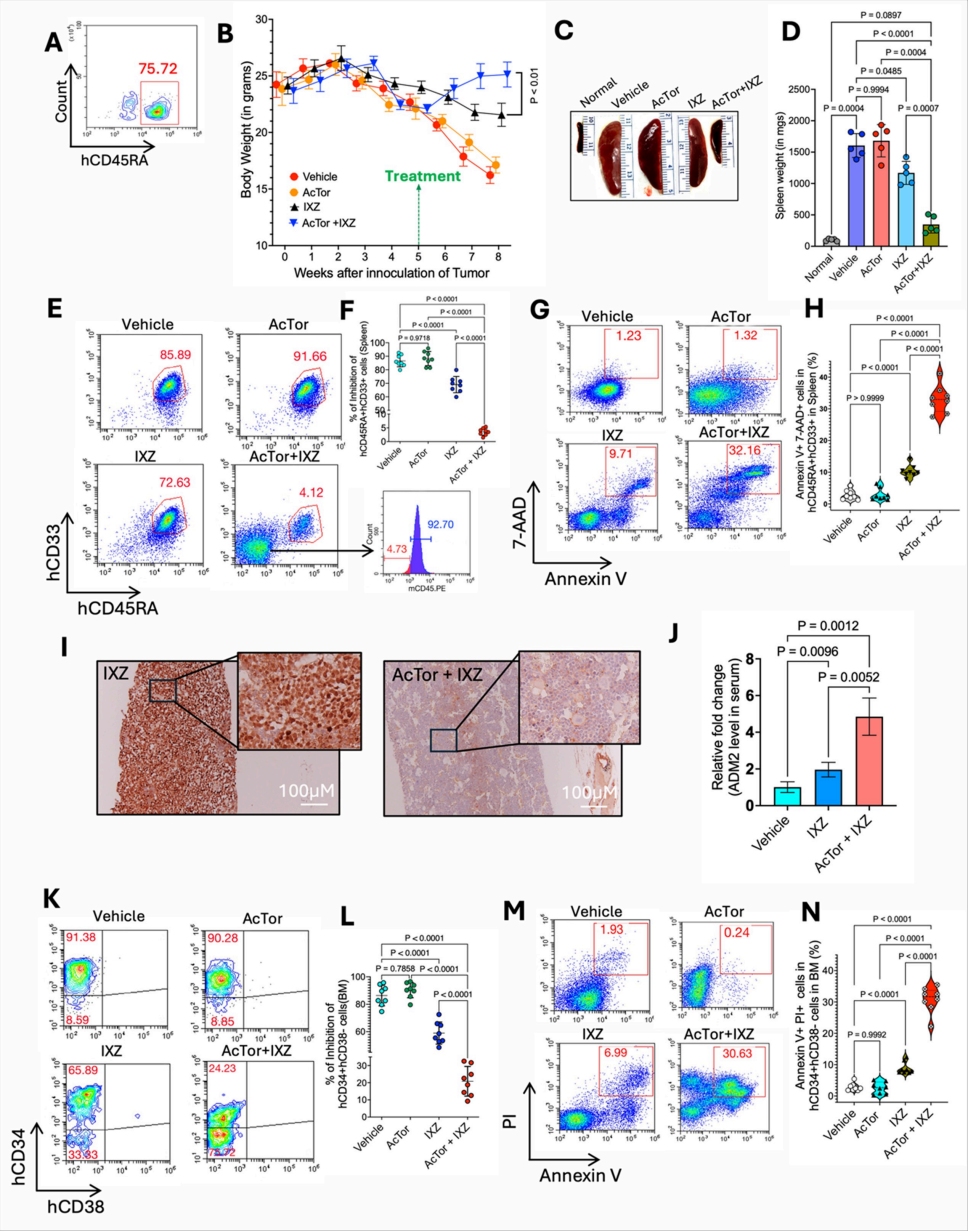
Treatment with AcTor/IXZ for three weeks induces apoptosis of patient-derived AML blasts and stem cells. **(A)** NSG mice were engrafted with patient-derived AML cells. When blood contained more than 75% of human CD45+ cells, treatment was initiated every other day for three weeks. **(B)** Measurement of body weight. **(C)** Typical spleen size after treatment and spleen weight distribution (**D**) (n=5). **(E)** Flow cytometry analyses of spleen cells for AML blasts and quantification. Only for AcTor/IXZ, mouse cells started to populate the spleen as evidenced by mCD45 staining. and quantification of the remaining (n=8) **(F)**Analysis of the CD45+/CD33+ AML cells for apoptosis using 7-AAD/Annexin V staining (n=8). **(G)** Typical immunohistochemistry images of bone marrow for Ki-67 from IXZ and AcTor/IXZ-treated mice. **(H)** Flow cytometry analyses of bone marrow for AML stem cells and quantification. Bone marrow AML cells reduced expression of the stem cell marker CD34 following AcTor/IXZ treatment (n=8). **(I)** Analysis of CD34+ AML cells for apoptosis using propidium iodide/Annexin V staining (n=8). **(J)** Relative levels of ADM2 in the serum of treated mice(n=5). Data are presented as mean ±SD. Statistical significance was determined using Brown-Frosythe and Welch one-way ANOVA followed by Dunnett’s T3 multiple comparisons test. Calculated *P*values are indicated in the graphs.

To address whether AcTor/IXZ is effective in eradicating AML stem cells, we challenged NSG mice with primary patient-derived AML (PDX), which were isolated from a FLT3-ITD mutated relapsed-refractory patient following Ara-C + daunorubicin treatment [48]. When injected into NSG mice, these PDX cells generated LSCs in the bone marrow, while blast cells are primarily in the periphery, mostly in the spleen. Treatment was initiated when human CD45RA+ cells exceeded 75% in the blood (Fig. 7A) and was limited to three weeks. At the endpoint, control mice were almost paralyzed. It was clear from the mouse’s appearance and activity that the combined treatment improved their condition (see movies). The weight of AcTor/IXZ-treated mice was increased within a week after treatment initiation (Fig. 7B), and spleen sizes were smaller (Fig. 7C, 7D). A strong reduction in the hCD33 + AML blast cells was observed with the emergence of a population of hCD33-negative, hCD45RA-negative, mouse CD45RA-positive cells (Fig. 7E, 7F). Analysis of the hCD45RA-positive spleen cells for apoptosis using Annexin V and 7-AAD showed that approximately 30% of the AML cells were in late apoptosis following AcTor/IXZ treatment (Fig. 7G, 7H). Gating strategies are shown in **Fig. S11**. Similar results were obtained for blast cells in the bone marrow and peripheral blood (**Fig. S12**).

Bone marrow AML cells at the late stages of the disease are positive for the proliferation marker Ki-67 [49]. A reduction in the Ki-67 positive nuclei of AcTor/IXZ-treated mice, compared to the other groups, was seen (Fig. 7I, **Fig. S13**). Analysis of serum ADM2 levels indicated an increase, providing further support for it as a marker for treatment (Fig. 7J). LSCs are enriched in the hCD34-positive, hCD38-negative population [49, 50]. AcTor/IXZ treatment reduced the hCD34-positive, hCD38-negative compartment (Fig. 7K, 7L), with an increase in apoptosis (Fig. 7M, 7N). We observed the accumulation of CD34/CD38 double-negative population in the AcTor/IXZ-treated cohort. This phenomenon was documented for cells treated with high concentrations of the PI bortezomib [36]. To examine if this is unique to the PDX cells, we treated KG-1a that also express the stem cell marker CD34 with AcTor/IXZ. The combined treatment also resulted in a reduction in CD34 expression (**Fig. S14A** and **B**), suggesting that CD34 expression is sensitive to the proteotoxic stress of PIs. We found no evidence of LSC maturation by CD11b, CD14 or CD15 markers (**Fig. S14**), indicating that LSC levels are reduced primarily by cell death. Masson’s trichrome staining of the bone marrow (tibia section) shows engraftment and localization of AML cells at the trabecular and cortical regions in vehicle treated mice (marked in yellow regions and arrows). These regions of the bone were cleared from AML cells in the AcTor/IXZ-treated mice (**Fig. S15**). Hypoxia supports the survival of LSCs stem cells [51, 52]. We assessed the level of bone marrow hypoxia by pimonidazole staining in the trabecular and cortical regions of the femur. In both locations, AcTor/IXZ treatment reduced hypoxic conditions (**Fig. S16**). We conclude that AcTor/IXZ combination is active by causing apoptosis of both AML blasts and LSCs.

Although the AML cells were barely detected after three weeks of treatment, five weeks after cessation of treatment, mice appeared sick again, indicating a relapse. Because PDX models of MM in mice show a rapid gain of resistance to proteasome inhibitors after relapse [53], we examined whether AcTor/IXZ treatment maintains potency. Mice were challenged with PDX cells, and when showing signs of sickness, were treated for three weeks with AcTor/IXZ. Then, after relapse, a cohort of mice was sacrificed (marked “Before”), and a cohort of mice was treated for the second time with AcTor/IXZ for three weeks and sacrificed (marked “After”, **Fig. S17A**). Comparison of spleen size before and after indicated a smaller size (**Fig. S17B**). hCD45RA+ cells were not detected after, coinciding with the emerging mouse CD45RA+ population (**Fig. S17C**). Most hCD45RA+ cells were Annexin V positive, with 15% at the late apoptotic stage (**Fig. S17D**). A smaller number of Ki-67-positive cells was seen after the second treatment (**Fig. 17E**) with a decrease in the hCD34+, hCD38− cells population (**Fig. S17F**), of those over 10% were apoptotic (**Fig. S17G**). We conclude that AcTor/IXZ maintains potency after relapse.

### AcTor/IXZ treatment shows efficacy for TP53-mutated PDX

**Figure 8:**
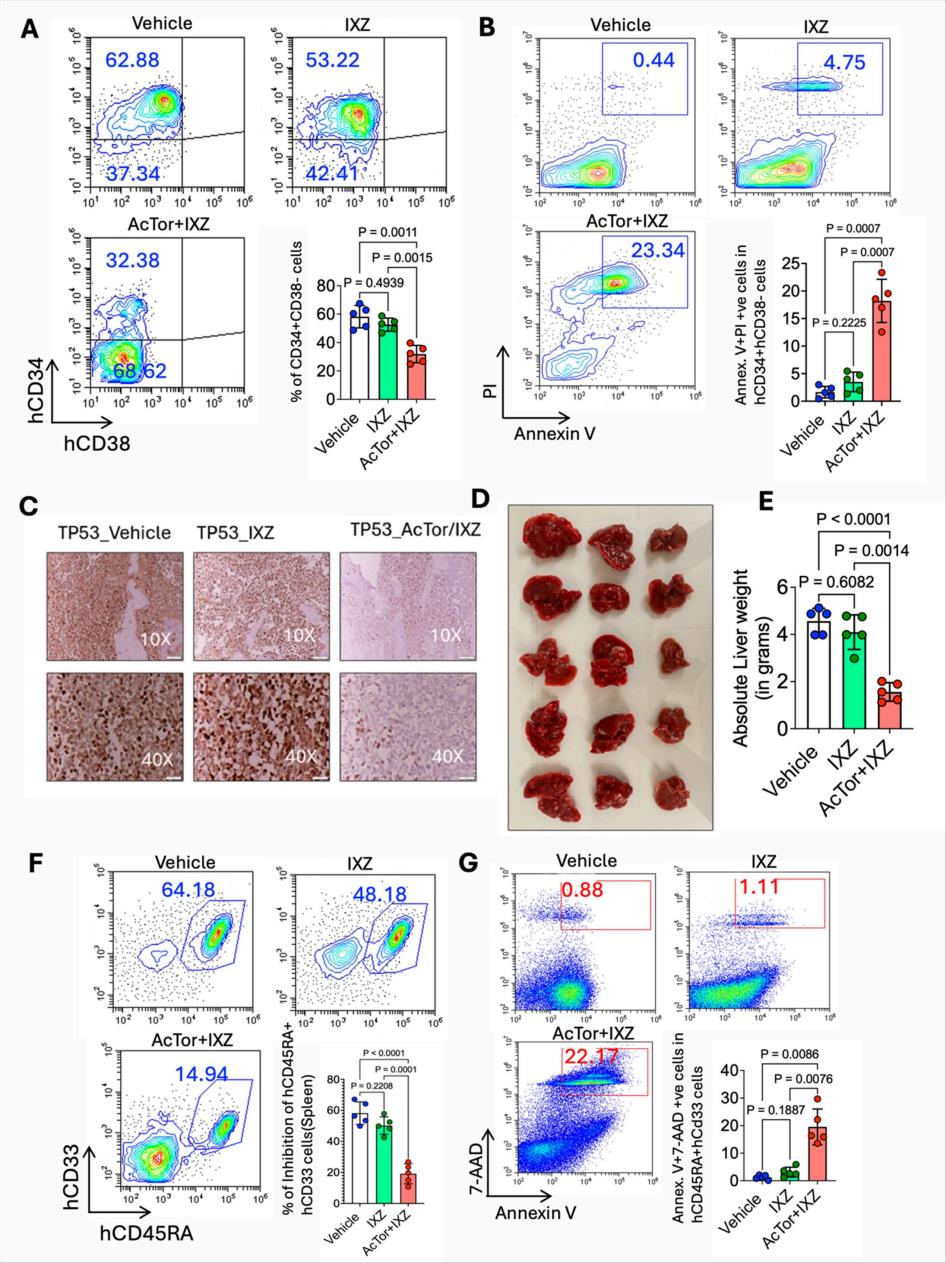
Treatment with AcTor/IXZ reduces the load of *TP53*-mutated patient-derived AML. NSG mice were engrafted with *TP53*-mutated patient-derived AML cells. Treatment was initiated two weeks after inoculation. Flow cytometry analyses of bone marrow were conducted for AML stem cells. **(A)** Bone marrow AML cells reduced expression of the stem cell marker CD34 following AcTor/IXZ treatment. **(B)** Analysis of CD34+ AML cells with propidium iodide/Annexin V staining indicated an increased apoptosis in AcTor/IXZ-treated mice. **(C)** Typical immunohistochemistry images of bone marrow for Ki-67 from vehicle, IXZ, and AcTor/IXZ-treated mice. **(D, E)** Livers were removed, weighed, and processed for histology (histology is shown in **Fig. S19**). **(F)** Flow cytometry analyses of spleen cells for AML blasts and quantification. Only for AcTor/IXZ, mouse cells started to populate the spleen as evident by mCD45 staining and quantification. **(G)** Analysis of CD33+/CD45+ AML cells for apoptosis using propidium iodide/Annexin V staining. Data are presented as mean ± SD from n=5 mice. Statistical significance was determined using Brown-Frosythe and Welch one-way ANOVA followed by Dunnett’s T3 multiple comparisons test. Calculated P values are indicated in the graphs.

**Figure 9:**
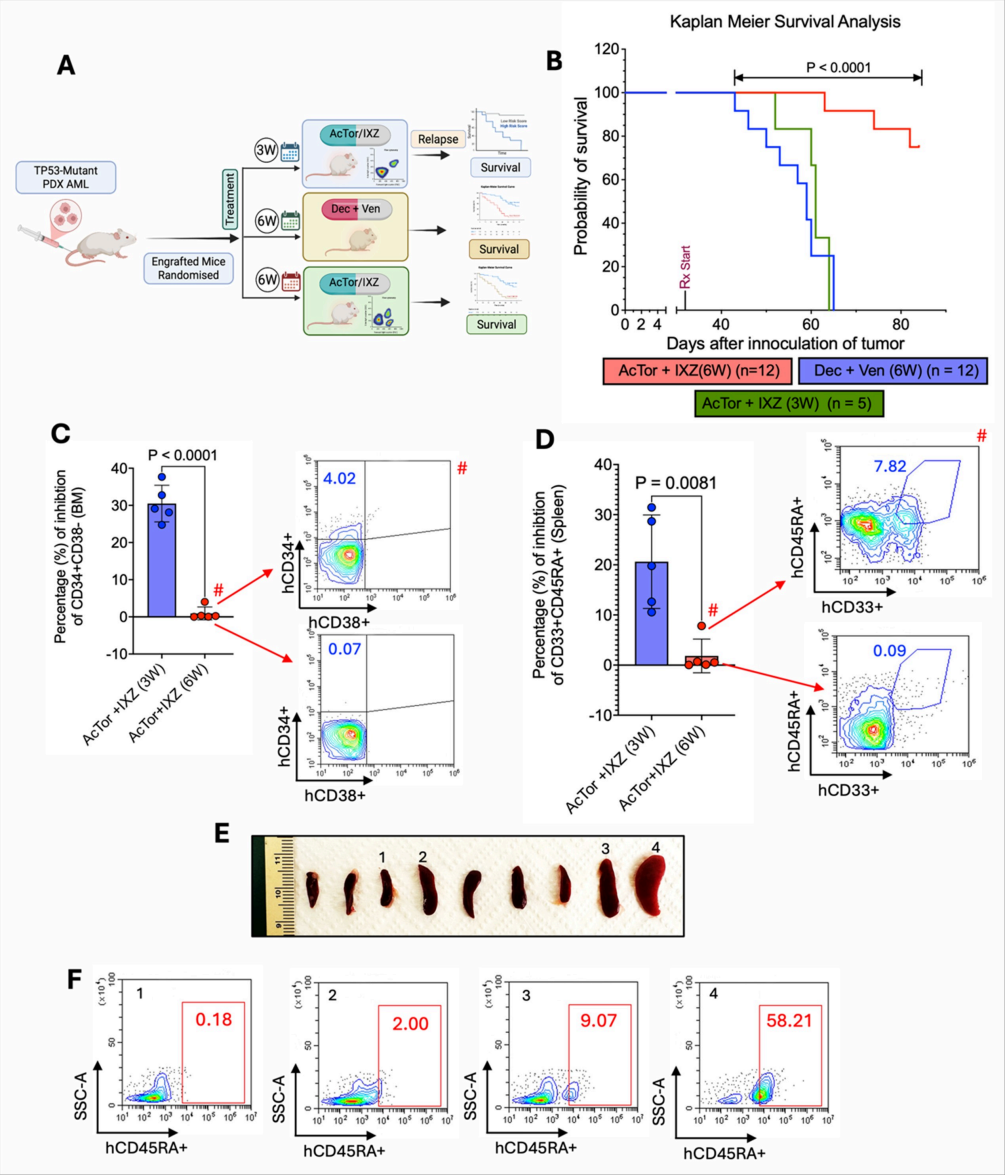
Prolonged Actor/IXZ treatment reduces AML burden of *TP53*-mutant PDX better than standard of care. **(A)**Schematic showing treatment schedules for AcTor/IXZ administered for 3 weeks or 6 weeks and Dec/Ven for 6 weeks following leukemic engraftment; **(B)**Survival analysis demonstrating significantly prolonged survival in mice receiving 6 weeks (n=12) of AcTor/IXZ therapy compared to the 3-week cohort after disease relapse (n=5) or Dec/Ven 6-week(n=12) cohorts. Survival curves were generated using the Kaplan-Meier method and compared using the log-rank (Mantel-Cox) tests and pairwise comparisons were adjusted using the Holm- Šídák method. **(C)**Quantitative analysis of leukemic stem cells from 3-week and 6-week AcTor/IXZ-treated mice. **(D)** Analysis of leukemic blasts from 3-week and 6-week treated mice after AcTor/IXZ therapy. Comparison of the two groups was done using a two-tailed unpaired t-test (n = 5). **(E)** Representative spleens are represented from AcTor/IXZ therapy three weeks after 6 weeks of therapy, which revealed the reduction of splenomegaly (none of the Dec/Ven treated group survived). **(F)** Representative flow-cytometric analysis of the CD45RA+ cells from AcTor/IXZ group reveals the disease reduction in <2% in seven out of nine mice that survived.

The efficacy of PIs in *TP53*-mutated myeloma and the activity in TP53-mutant MV4–11 prompted us to test whether AcTor/IXZ is effective for treating *TP53*-mutated patient AML. NSG mice were engrafted with PDX derived from an AML patient suffering from *TP53*/*Cbl* double mutant tumor, representing a rare and highly aggressive type [54, 55]. Owing to the aggressiveness of the model, we initiated the treatment 10 days after inoculation. To assess efficacy, mice were treated for three weeks with vehicle, IXZ, and AcTor/IXZ. After three weeks, the mice in the control and IXZ groups were hardly moving, while the mice in the AcTor/IXZ group looked normal (see films in **Fig. S18**). Upon sacrifice, analysis of the bone marrow showed a similar effect as observed for *TP53* WT PDX. Expression of CD34 was reduced in the AcTor/IXZ treated mice (Fig. 8A). A larger proportion of the CD34-positive cells in AcTor/IXZ treated mice were apoptotic (Fig. 8B). Ki67 positive cells in the bone marrow were reduced (Fig. 8C). We noticed that spleens were moderately enlarged and the tumor cells mostly infiltrated the liver, generating nodules and an increase in total liver mass. Mice treated with AcTor/IXZ had smaller livers with fewer nodules (Fig. 8D, 8E). Histological analyses of the livers showed that most of the liver was occupied with AML cells in all groups besides the AcTor/IXZ ones (**Fig. S19**). Analysis of the spleens demonstrated less blast CD33-positive cells (Fig. 8F) and a larger proportion of apoptotic Annexin V-positive cells (Fig. 8G). We conclude that AcTor/IXZ is also effective in *TP53*-mutated AML. We observed that three weeks of treatment were insufficient to fully eliminate AML cells. We therefore repeated the experiment, this time extending the treatment to six weeks. As a clinical reference, we included a cohort treated with a combination of the hypomethylating agent decitabine (Dec) and BCL2 inhibitor Venetoclax (Ven) (Fig. 9A), a regimen commonly used for TP53-mutated AML [6]. Ten days after the cessation of the three-week treatment of AcTor/IXZ, mice started to succumb. A subset of mice also succumbed during Dec + Ven therapy. In contrast, all mice treated with AcTor/IXZ were alive during the treatment (Fig. 9B). When all mice treated with Dec + Ven died, five mice from AcTor/IXZ cohort were sacrificed to assess tumor burden and compared to the data of the 3-week treatment. Four of the five mice treated for 6 weeks exhibited minimal tumor burden of less than 1% of total cells in both bone marrow (Fig. 9C) and spleen (Fig. 9D). The remaining mice were monitored for an additional three weeks. Of the 12 remaining mice, 9 survived to the end of the study (Fig. 9B). Mice were sacrificed and spleens were removed (Fig. 9E) and analyzed for AML blasts, which revealed that seven of nine surviving mice carried a tumor burden below 2%, while a few had no detectable AML cells (spleen 1 in Fig. 9F). We conclude that AcTor/IXZ in this model is superior to Dec + Ven and can bring a TP53-mutated PDX to an undetectable level after 6 weeks of treatment.

## Discussion

PIs exert their anti-cancer activity by perturbing proteostasis. Resistance to PIs employs cell intrinsic and tumor microenvironment-dependent mechanisms [56]. The large number of mechanisms attributed to resistance to PIs makes it a challenge to address therapeutically. However, an immediate adaptation to the proteostatic stress is essential. Based on our previous findings, the suppression of mTORC1 is part of this immediate response, to PIs and can be a gateway to resistance [22]. We hypothesized that if high mTORC1 activity is imposed during the initial exposures to PIs, resistance will be delayed if not prevented. Incentivized by this hypothesis, we generated AcTor.

Among the three clinically approved PIs, we selected Ixazomib (IXZ) for these studies because of its oral bioavailability and prolonged half-life. AcTor was derived from cobicistat and is therefore predicted to retain favorable pharmacologic properties, including oral absorption, raising the possibility that both compounds could ultimately be administered in a combined formulation. *In vitro*, AcTor promotes the activity of IXZ in AML cells independently of specific mutations, including *TP53-*altered contexts [14]. The combination of AcTor and IXZ elicited irreparable damage to mitochondrial ETC, including complex IV. This mechanism was shown for IXZ in TSC2 KO MM cells [22], and in TSC2-silenced AML cells. Together with indirect evidence of binding to TSC2, our data support that AcTor primarily operates by blocking TSC2. Since all mitochondrial ETC complexes are embedded in the mitochondrial inner membrane [57], AcTor/IXZ may cause a defect in mitochondrial transport. A mild impairment in protein import to the mitochondria or low stability of one of the complex components can result in ETC insufficiency. Proteomic analyses suggested that mitochondrial components, including components of the ETC, are subjected to a low level of ubiquitination [58]. It is therefore possible that in the presence of AcTor and IXZ, respiratory complexes accumulate ubiquitination to a level that perturbs proper function. Additionally, elevation of mitochondrial oxidative phosphorylation at the expense of glycolysis is one of the cellular strategies to adapt to PIs [59]. By suppressing oxidative phosphorylation, the insult to the mitochondria may invoke a feedforward response that results in a collapse of mitochondrial respiration.

AML cells rely on PI3K signaling for survival, and inhibition of mTOR in the presence of chemotherapy impairs survival [60]. However, to achieve a durable anti-cancer effect, mTOR should be suppressed to a level that cannot be tolerated by most patients [61]. A reporter mouse for mTOR activity showed a biphasic behavior of mTOR during AML progression. mTOR activity was reduced with the initial AML progression, while induced later, even in the presence of treatment [62]. We were therefore concerned that an mTOR inducer might accelerate AML growth. Transcriptome analyses indicate that on the background of IXZ, AcTor does not share the expression signature when added alone. In fact, AcTor in the presence of IXZ, fortified stress signaling pathways that are consistent with anti-cancer responses.

In support, when given alone, AcTor slightly accelerated disease progression. The pharmacokinetic properties of IXZ are optimal for combination with AcTor. The half-life of IXZ is estimated in days, allowing its administration once a week to MM patients. While the pharmacokinetic parameters of AcTor have not been determined, the cobicistat half-life is approximately 3 h [63]. We therefore project that if given together on a once-a-day basis, IXZ will generate a stable, steady state concentration, while AcTor will generate spikes in serum concentration and be cleared within 12–24 h. This should ensure that tumor cells will never be exposed to AcTor alone.

All AML therapies compromise hematopoiesis. The major safety concern of AML patients is the ability to rapidly restore bone marrow functions following treatment. This relies on sparing enough HSCs. Normal adult mice have between 5,000–10,000 long-term HSCs, comprising 0.01% of total bone marrow cells [64]. However, transfer experiments show that one hundred HSC is sufficient to replenish hematopoiesis [65]. Encouraged by the fact that following treatment with AcTor/IXZ the murine HSCs were reduced by 30% at most, displaying normal blood counts, we conclude that AcTor/IXZ treatment should not cause irreversible immunosuppression. This is probably due to reliance on glycolysis by HSCs [66], making them less sensitive to mitochondrial damage.

During remission, AML LSCs acquire mutations that result in a much more aggressive disease upon relapse [67]. In addition to the canonical resistance mechanisms, such as overexpression of P-glycoprotein, glutathione S-transferases, and activation mutations in key prosurvival pathways, AML adjusts cellular respiration, enhances autophagy, and modifies energy sources [68]. We recently found that resistance of AML to venetoclax, a Bcl-2 inhibitor, is associated with ATP hydrolysis [41]. Importantly, AcTor/IXZ combination remained effective across multiple Venetoclax-resistant cell lines. Notably, AcTor alone reduced viability in these models, suggesting that activation of mTOR signaling is detrimental to cells that have adapted their mitochondrial metabolism in response to Venetoclax. Furthermore, the combination retained its activity following the disease relapse, without the need of dose escalation. These findings indicate that AcTor/IXZ may provide a therapeutic option for AML that has progressed beyond the currently available treatment strategies.

Despite novel therapies for AML, *TP53*-mutated AML represents a subgroup that has failed to improve, with an overall survival of ~6 months that is independent of age and fitness. We did not observe a reduced efficacy of AcTor/IXZ even in one of the most aggressive AML models. Extension of the treatment resulted in a complete disappearance of the tumor cells in some of the mice. Importantly, AcTor/IXZ treatment superseded the combination of decitabine and venetoclax, which is considered the standard of care for *TP53*-mutated AML. It remains to be determined whether AcTor will be useful when combined with additional anti-AML drugs and how it affects LSC viability after a gain of resistance to chemotherapy. Further analysis is needed to compare AcTor/IXZ to an optimized drug regimen in a larger number of *TP53*-mutated and TP53-wild-type AML for further clinical development.

## Supplementary Material

This is a list of supplementary files associated with this preprint. Click to download.

- Supplemetaryfigures.pdf
- Supplementalmaterialandmethods.docx
- AcTorPaperuncroppedWB.xlsx

## Data Availability

Bulk RNA sequencing data is available through https://www.ncbi.nlm.nih.gov/bioproject/PRJNA1234706. Other data are available in supplementary figures (Fig. S1 to Fig S19). Movies of AML-engrafted mice after treatments are provided in Fig. S18. Uncropped western blots used in this study are provided in supplementary material. Any additional information will be provided upon request.
